# GOPET: A tool for automated predictions of Gene Ontology terms

**DOI:** 10.1186/1471-2105-7-161

**Published:** 2006-03-20

**Authors:** Arunachalam Vinayagam, Coral del Val, Falk Schubert, Roland Eils, Karl-Heinz Glatting, Sándor Suhai, Rainer König

**Affiliations:** 1 Department of Molecular Biophysics, German Cancer Research Center (DKFZ), Im Neuenheimer Feld 580, 69121 Heidelberg, Germany; 2 Division of Theoretical Bioinformatics, German Cancer Research Center (DKFZ), Im Neuenheimer Feld 580, 69121 Heidelberg, Germany; 3 Department of Bioinformatics and Functional Genomics, Institute for Pharmacy and Molecular Biotechnology, University of Heidelberg, 69120 Heidelberg, Germany

## Abstract

**Background:**

Vast progress in sequencing projects has called for annotation on a large scale. A Number of methods have been developed to address this challenging task. These methods, however, either apply to specific subsets, or their predictions are not formalised, or they do not provide precise confidence values for their predictions.

**Description:**

We recently established a learning system for automated annotation, trained with a broad variety of different organisms to predict the standardised annotation terms from Gene Ontology (GO). Now, this method has been made available to the public via our web-service GOPET (Gene Ontology term Prediction and Evaluation Tool). It supplies annotation for sequences of any organism. For each predicted term an appropriate confidence value is provided. The basic method had been developed for predicting molecular function GO-terms. It is now expanded to predict biological process terms. This web service is available via

**Conclusion:**

Our web service gives experimental researchers as well as the bioinformatics community a valuable sequence annotation device. Additionally, GOPET also provides less significant annotation data which may serve as an extended discovery platform for the user.

## Background

The expanding amount of sequence data generated from genome and cDNA sequencing projects creates an ever extending demand for automated annotation. The annotation represented in standardised formats like the ones designed by ontologies benefits from its straightforward operability across different analysis platforms. The Gene Ontology (GO) project is a collaborative initiative and provides consistent descriptions of gene products across different species [1,2]. This gives Gene Ontology the potential of becoming a major basis for automatic annotation.

Gene product prediction is confronted with a variety of challenges coming from ambiguities concerning the underlying input databases, e.g. sequence errors, erroneous and incomplete annotation, and inconsistent annotation across databases or consistent but erroneous annotation across databases. A broad variety of excellent annotation systems have been developed to tackle these problems, e.g. RiceGAAS [3], GAIA [4], Genotator [5], Magpie [6], GeneQuiz [7], GeneAtlas [8], PEDANT [9], cDNA2Genome [10], GenDB [11], GOFigure [12] and GOtcha [13]. However, little has been done to quantify the annotation accuracy by defined benchmarks *and *establishing a method to provide a confidence value for each annotation. We developed an automated system for large-scale cDNA function assignment, designed and optimised to achieve a high-level of prediction accuracy without any manual refinement. With our system, Gene Ontology molecular function terms are predicted for uncharacterised cDNA sequences and a defined confidence value is calculated for each prediction. The performance of the system was benchmarked with 36,771 GO annotated cDNA sequences derived from 13 organisms [14].

We have now extended our approach to predict biological process terms and implemented our method as an online sequence annotation tool (GOPET, Gene Ontology term Prediction and Evaluation Tool). From a user-friendly front-end, the user can upload query protein- and nucleotide-sequences for which the tool assigns Gene Ontology molecular function and biological process terms. It is implemented under the W3H-Task-System which provides a flexible way to configure program and data flow between different biocomputational methods [15]. The W3H-Task-System uses the Heidelberg Unix Sequence Analysis Resources (HUSAR [16]) which is a sequence analysis software package operating at the German Cancer Research Center.

## Construction and content

Nucleotide or protein query sequences are blasted [17] against GO-mapped protein databases. Currently, we apply 16 GO-annotated protein databases from the following model organisms (downloaded from the data sources given in brackets): *Saccharomyces cerevisiae *(Stanford University), *Drosophila melanogaster *(Berkeley Drosophila Genome Project), *Mus musculus *(Ensembl), *Arabidopsis thaliana *(MIPS), *Caenorhabditis elegans *(Sanger Center), *Rattus norvegicus *(NCBI), *Danio rerio *(SwissProt), *Leishmania major *(Sanger Center), *Bacillus anthracis Ame *(TIGR), *Coxiella burnetii RSA 493 *(NCBI), *Shewanella oneidensis MR-1 *(TIGR), *Vibrio cholerae *(TIGR)*, Plasmodium falciparum *(Plasmodium Genome Research), *Oryza sativa *(SwissProt, Trembl), *Trypanosoma brucei *(Sanger Center), *Homo sapiens *(GOA annotated sequences of SwissProt, Trembl and Enseml), as well as the protein database SwissProt (the SwissProt part of the UniProt family of databases). These databases are constantly updated to keep track of the latest information. The corresponding GO annotations were taken from Gene Ontology [18]. The Sequence Retrieval System SRS [19] is used to retrieve GO annotation from GO-mapping relations. Blast hits with a relaxed e-value threshold (e-value < 0.01) are considered and all GO-terms are extracted from these hits. Each extracted GO-term is attached to a broad variety of elaborated attributes, including sequence similarity measures, such as e-value, bitscore, identity, coverage score and alignment length. Further attributes use GO-term frequency, GO-term relationships between homologues, the level of annotation within the GO hierarchy and annotation quality of the homologues which are calculated using the evidence codes provided by the gene association tables of the GO mapped sequence databases. Nine commonly used evidence codes are taken into acoount: TAS, NAS, ISS, IPI, IMP, IGI, IEP, IEA, IDA. The entries of theses attributes for each GO term are calculated by summing over the occurrences of the corresponding evidence codes of all blast hits (for more details, see [14]). In the following, the term "instance" is used for a GO-term together with its corresponding attribute values.

### Training

For training and testing the SVM, we selected 39,740 GO-annotatedcDNA sequences from the following organisms: *Saccharomyces cerevisiae*, *Drosophila melanogaster*, *Mus musculus*, *Arabidopsis thaliana*, *Caenorhabditis elegans*, *Rattus norvegicus*, *Danio rerio*, *Leishmania major*, *Bacillus anthracis Ame*, *Coxiella burnetii RSA 493*, *Shewanella oneidensis MR-1*, *Vibrio cholerae *and *Plasmodium falciparum *(same database sources as for the protein sequences). During the training phase, each instance is compared to the GO annotation of the (known) query sequence. It is classified as "correct" if the GO-term of the instance corresponds to one of the GO-terms from the query sequences, and labelled as "false" otherwise. Support Vector Machines (SVM) are applied to determine the separation between "correct" and "false" instances. Support Vector Machines were chosen due to their ability to learn any decision function [20]. Furthermore, Support Vector Machines have shown a very good generalisation performance, both from a theoretical [21] and empirical point of view [22].

### Application

After training, the classifier is able to select GO-terms for an unknown query sequence by the same procedure: the query sequence is blasted against the annotated protein sequences of the database, GO-terms from the hits are extracted together with their corresponding attribute values. This instance is transferred to the SVM and classified in accordance to its attribute values.

Note, that we yielded a high amount of instances for training (856,632 instances). Therefore, we could apply a voting scheme. This consists of an assembly of 99 classifiers corresponding to ≈8,600 training instances each. So, multiple classifiers are employed for the classification. The predicted results are combined by a committee approach [23] in which each classifier contributes a vote that predicts a particular instance as correct. All classifiers got the same weight. Note, that our voting scheme compares to bagging methods in which all classifiers are given the same weight [24]. The number of votes are summed up for each instance. A confidence value is calculated for each GO-term by comparing the number of votes with the corresponding number of true positives divided by all positives during the testing phase. The system was benchmarked by an organism-wise cross-validation, i.e. a set of (known) sequences with GO annotations was chosen to train the classifier. This set contained sequences for all except one organism. Then the performance was tested with the sequences of the remaining organism. This was done for all selected organisms and the overall prediction performance was calculated (for details, see [14]).

## Biological process prediction

We applied the same approach to predict biological process terms and trained 99 new SVM classifiers specifically on GO-terms for biological process. GO-terms for each blast hit were extracted by considering GO-terms corresponding to biological process and by discarding GO-terms that were prefixed with NOT (annotators state that a particular gene product is NOT associated with a particular GO term), or corresponding to "biological process unknown". We were able to select 27,109 sequences from 13 model organisms for training and validation and yielded 1,342,270 instances. Therefore, each classifier was trained with ≈13,558 instances. Table 1 shows the prediction performances for molecular function and biological process. We got only 12% of positive instances for training and testing. When compared to molecular function (31% positive instances), inferring biological process from the sequence was less often possible. This shows that our tested nucleotide sequences encode more information on their molecular function than their biological process. However, the validation result showed a high recall (76%) for biological process terms at 80% precision.

## Comparison with GOtcha and GOFigure

We compared our system with the well established annotation tools GoFigure [12] and GOtcha [13]. GoFigure performs a homology search and constructs the minimum covering graph from the extracted GO terms. The terms are scored based on Blast e-values and terms above a defined threshold are given out. GOtcha predictions are supplied with confidence values (P-score). Basically, these P-scores are calculated from three different scores which are derived from sequence similarity and the GO structure. We took eight *Xenopus laevis *contig sequences, which we annotated with our system previously [14] to compare our results for molecular function to GoFigure and GOtcha (using default parameters). The results are shown in Table 2. For GOPET and GOtcha the same confidence threshold of 80% was applied. In general, all three methods show very similar predictions. However, for TC190605 GoFigure yielded a conflicting result (nuclease activity) when compared to GOPET (serine hydrolase activity), while GOtcha supported a more general term (Enzyme activity). GOPET and GOtcha predictions were highly comparable, though the specificity of the annotation varied for some cases. For example, GOtcha provided more specific terms for TC212171, TC187949 and TC199713. For TC196381, TC209487, and TC190605 GOPET annotated more specific terms. For TTC194305 GOTcha didn't get any results with the defined threshold. Interestingly, both system predicted TC210151 as frizzled receptor with similar confidence values (GOPET 82% and GOtcha 80%).

Furthermore, we compared GOPET and GOTCHA in more detail. We selected manually 100 random sequences (excluding IEA annotated ones) from DictyBase (Version 1.68, DictyBase, database for *Dictyostelium discoideum*[25]). This database was used for the comparison as it was not used for the training of both systems. From GOPET and GOTcha, we selected again only annotations with ≥ 80% confidence. Of the 100 sequences, 72 and 77 were annotated with 332 (true positive: 296) and 535 (true positive: 430) terms by GOPET and GOTcha, respectively. Hence, GOPET showed a better precision (89%) compared to GOTcha (80%) (p-value of a two-sided Fisher exact test: 0.0006) and GOTcha showed a better recall. Note that, in the most cases, GOPET provided more specific annotation terms (all data is provided in supplementary Tables S1 and S2, see Additional file 1 and Additional file 2, respectively).

## Implementation

**Figure 1:**
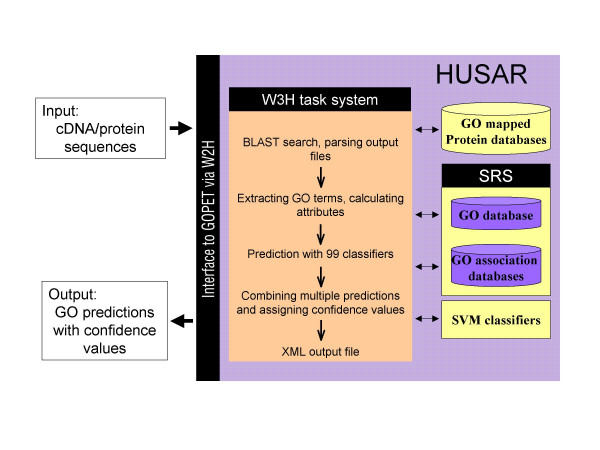
General workflow of the GOPET web-server.

The W3H task framework has been developed in the HUSAR environment at the German Cancer Research Center [15]. HUSAR includes the tools of the GCG [26], EMBOSS [27] packages and the Phylip suite [28] as well as many other applications. Common sequence databases are also available in the HUSAR environment. The W3H task framework allows the integration of applications and methods to create tailor-made task flows, which can be used in high throughput analyses without the usual need for customised programming. By specifying the program flow and dependency rules between the used applications in the meta-data, tasks of high complexity can be designed. The integration of W3H into W2H (graphical web interface to HUSAR [29,30]) allows tasks like GOPET to be easily made available on the web without additional programming. The GO database and all sequence to GO-mapping information are implemented in SRS and accessed through an SRS-API. The LIBSVM package ([31], version 2.4) is installed in HUSAR and was used for our classifications. Figure 1 illustrates the general workflow of the system.

## Utility and discussion

**Figure 2:**
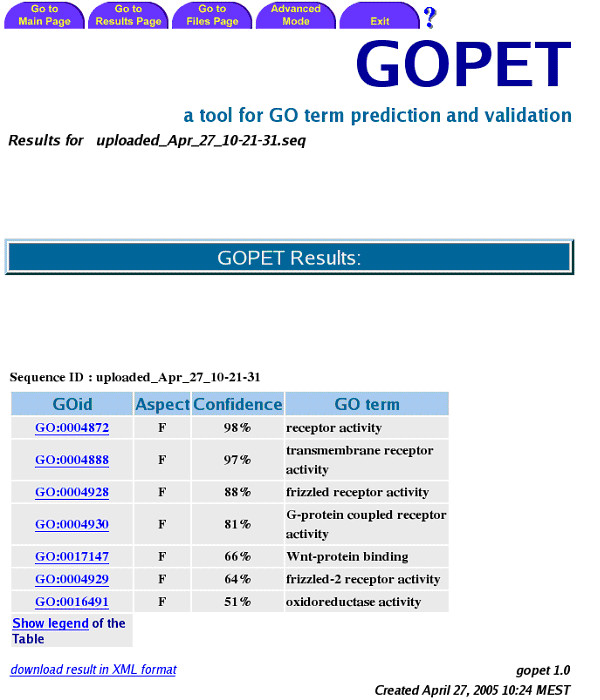
Example output of GOPET: the first column shows the GO ID, followed by its aspect (molecular function), the confidence value of the prediction and the short description of the GO-term. The example here shows the query results for sequence Xfz4 (Xenopus frizzled 4). Note, that Xfz4 is a maternal mRNA whose carboxyl-terminal half contains putative transmembrane segments. Furthermore, it is homologous to the murine gene product Mfz4, a frizzled transmembrane protein [34]. It has been shown elsewhere, that the C-terminal cytoplasmic Lys-thr-X-X-X-Trp motif in frizzled receptors mediates Wnt/beta-catenin signalling [35].

The GOPET web-server is accessible via the web-page [32]. The starting page shows the user interface to upload query sequences. More than one sequence can be queried if uploaded in FastA format. GOPET accepts nucleotide and protein sequences as input. The running time of the task depends on the size and complexity of the input sequences and varies between 2 to 4 minutes per sequence on our Sun Multiprocessor machine (Sun E4500). For example, annotating a cDNA sequence with 1173 base pairs (TC291942 from TIGR XGI, [33]) takes about 2 min 30 seconds. Figure 2 shows the prediction results for this sequence. The output displays every predicted GO-term having at least one vote. The results are sorted by their confidence values. By default, only the top 20 predictions are displayed if more hits have been found.

Confidence values may serve in several ways. Predictions with confidence values ≥ 80% can be used straight away for annotation. In contrast, predictions with low confidence values may serve as a basis for new hypotheses and research, e.g. to infer further relationships to the original function. Automated annotation fails for sequences without any annotated and known homologues and the only alternative remains to analyse the sequence manually and in depth. We included IEA annotated sequences (automated annotated sequences) to improve the annotation coverage. To compare the performance with and without IEA annotated sequences, we calculated the respective prediction accuracies for yeast (non-IEA) based on the worm data-set (IEA) and fly data-set (non-IEA). The results were quite similar (test yeast and training worm: 82%, test yeast and training fly: 81% accuracy, see [14] for details). However, restricting to non-IEA terms may improve the precision. We consider to provide an option for the user to exclude IEA annotations based predictions for the next release of GOPET.

## Conclusion

GOPET serves as a valuable tool for experimental researchers as well as for the bioinformatics community. The underlying methodology shows numerous advantages. The prediction performance is organism-independent, since the applied annotation databases cover a broad variety of different organisms and the attributes selected for classification are by definition not specific to any organism. The prediction quality can be assessed by assigned confidence values. It could be shown recently, that the prediction quality for confidence values ≥ 80% is comparable to high-quality manual annotation [14]. Our confidence values give the user a concrete evaluation of the results and a distinct further processing capability when inspecting the annotation at different levels of certainty. The GOPET server predicts both molecular function and biological GO-terms. In future, we plan to predict cellular component terms and to improve the quality of biological process predictions by including protein-protein interaction data.

## Availability

GOPET is available via Open-HUSAR, (Heidelberg Unix Sequence Analysis Resources) [32].

Contact: genome@dkfz.de

## Authors' contributions

All conceived the idea. AV carried out the work and drafted the manuscript. CD implemented the program into the W3H system. RK and FS contributed in developing the methodology and drafting the manuscript. KG implemented the databases in SRS. RK, SS, KG and RE supervised the work. All authors participated in reading, approving and revising the manuscript.

## Supplementary Material

Additional File 1 Table S2. Comparison of GOPET with the annotation of 100 random selected protein sequences of *Dictyostelium discoideum* Click here for file

Additional File 2 Table S2. Comparison of GOTcha with the annotation of 100 random selected sequences of *Dictyostelium discoideum* Click here for file

## Figures and Tables

**Table 1:** Comparison of the prediction performance for molecular function and biological process.

	Molecular function	Biological process
Number of sequences used for SVM training and validation	36,771	27,109
Number of instances used in training and validation sets	856,632	1,342,270
Positive instances	31%	12%
Recall at 80% precision	65%	76%

**Table 2:** Comparison of our system (GOPET) with the annotation systems GOtcha and GOFigure to predict the molecular function for eight *Xenopus leavis *sequences. Basically, the first four hits are shown, for GOPET and GOtcha with confidence values ≥ 80%.

	GOPET			GOtcha			GOFigure	
Contig	GO ID	Confidence	GO term	GO ID	Estimated likelihood	GO term	GO ID	GO term

TC212171	0008233	100%	peptidase activity	0003824	98%	enzyme activity	0004263	chymotrypsin activity
	0004175	100%	endopeptidase activity	0008233	98%	peptidase activity	0004295	trypsin activity
	008236	98%	serine-type peptidase activity	0016787	98%	hydrolase activity		
	0016787	98%	hydrolase activity	0008236	98%	serine-type peptidase activity		
TC196381	004175	100%	endopeptidase activity	0003824	93%	enzyme activity	0004263	chymotrypsin activity
	0016787	98%	hydrolase activity				0004295	trypsin activity
	0008233	98%	peptidase activity					
	0008236	97%	serine-type peptidase activity					
TC209487	0003824	100%	enzyme activity	0003824	90%	enzyme activity	0004177	aminopeptidase activity
	0016787	100%	hydrolase activity				0004301	epoxide hydrolase activity
	0004177	90%	aminopeptidase activity					
	0017171	85%	serine hydrolase activity					
TC187949	0004888	100%	transmembrane receptor activity	0004872	93%	receptor activity	0004888	transmembrane receptor activity
	0004872	97%	receptor activity	0004888	93%	transmembrane receptor activity		
				0004871	93%	signal transducer activity		
				0004930	93%	G-protein coupled receptor activity		
TC194305	0003824	100%	enzyme activity	-	-	-	0004674	protein serine/threonine kinase activity
	0016740	99%	transferase activity				0005524	ATP binding
	0016301	99%	kinase activity					
	0004672	97%	protein kinase activity					
TC210151	0004872	100%	receptor activity	0004872	98%	receptor activity	0004926	non-G-protein coupled 7TM receptor activity
	0004888	97%	transmembrane receptor activity	0004871	98%	signal transducer activity	0004930	G-protein coupled receptor activity
	0004928	82%	frizzled receptor activity	0004888	98%	transmembrane receptor activity		
				0004930	98%	G-protein coupled receptor activity		
				0004926	92%	non-G-protein coupled 7TM receptor activity		
				0004928	80%	frizzled receptor activity		
TC199713	0004602	100%	glutathione peroxidase activity	0003824	99%	enzyme activity	0004601	peroxidase activity
	0016491	98%	oxidoreductase activity	0004601	99%	peroxidase activity	0004602	glutathione peroxidase activity
	0004601	85%	peroxidase activity	0016491	99%	oxidoreductase activity		
				0016684	99%	oxidoreductase, acting on peroxide as acceptor activity		
				0004602	97%	glutathione peroxidase activity		
TC190605	0003824	100%	enzyme activity	0003824	92%	enzyme activity	0004518	nuclease activity
	0016787	100%	hydrolase activity					
	0017171	87%	serine hydrolase activity					
