# Supplementary material for: GOPET: A tool for automated predictions of Gene Ontology terms
Source: BMC Bioinformatics. 2006 Mar 20;7:161. doi: 10.1186/1471-2105-7-161 (PMC1434778; doi:10.1186/1471-2105-7-161)
Supplement: Additional File 1 — Table S2. Comparison of GOPET with the annotation of 100 random selected protein sequences of Dictyostelium discoideum [file 1471-2105-7-161-S1.doc]

**Table S1**

Comparison of GOPET with the annotation of 100 random selected protein sequences of *Dictyostelium discoideum* (Http://DictyBase.org). Column three shows the prediction results (only predictions with a confidence value ≥ 80% are shown). Column four shows the results of the comparisons to the annotation of DictyBase (T = true, F = False).

| running number | sequence id | our predictions | comparison to DictyBase |
| --- | --- | --- | --- |
| 1 | DDB0185426 | GO:0016787 | T |
| 1 | DDB0185426 | GO:0016798 | T |
| 1 | DDB0185426 | GO:0004553 | T |
| 1 | DDB0185426 | GO:0008810 | T |
| 2 | DDB0218878 | GO:0016740 | T |
| 2 | DDB0218878 | GO:0016301 | T |
| 2 | DDB0218878 | GO:0004672 | T |
| 2 | DDB0218878 | GO:0005024 | T |
| 2 | DDB0218878 | GO:0004714 | T |
| 2 | DDB0218878 | GO:0004681 | F |
| 2 | DDB0218878 | GO:0005025 | F |
| 2 | DDB0218878 | GO:0004692 | F |
| 2 | DDB0218878 | GO:0004697 | T |
| 2 | DDB0218878 | GO:0004698 | T |
| 2 | DDB0218878 | GO:0004701 | T |
| 2 | DDB0218878 | GO:0004705 | T |
| 2 | DDB0218878 | GO:0004709 | T |
| 2 | DDB0218878 | GO:0004675 | T |
| 2 | DDB0218878 | GO:0005015 | T |
| 2 | DDB0218878 | GO:0004682 | T |
| 2 | DDB0218878 | GO:0004687 | F |
| 2 | DDB0218878 | GO:0016909 | F |
| 2 | DDB0218878 | GO:0017002 | F |
| 2 | DDB0218878 | GO:0005026 | F |
| 3 | DDB0232367 | GO:0004003 | T |
| 3 | DDB0232367 | GO:0003678 | T |
| 4 | DDB0191196 | GO:0005509 | T |
| 4 | DDB0191196 | GO:0005488 | T |
| 5 | DDB0191161 | - | - |
| 6 | DDB0216392 | - | - |
| 7 | DDB0232344 | GO:0003824 | T |
| 7 | DDB0232344 | GO:0004497 | T |
| 7 | DDB0232344 | GO:0008387 | F |
| 7 | DDB0232344 | GO:0004507 | T |
| 7 | DDB0232344 | GO:0004508 | T |
| 7 | DDB0232344 | GO:0016491 | F |
| 7 | DDB0232344 | GO:0008393 | F |
| 7 | DDB0232344 | GO:0008396 | F |
| 7 | DDB0232344 | GO:0008389 | F |
| 7 | DDB0232344 | GO:0008401 | F |
| 7 | DDB0232344 | GO:0004509 | T |
| 7 | DDB0232344 | GO:0008392 | T |
| 7 | DDB0232344 | GO:0004498 | T |
| 7 | DDB0232344 | GO:0008123 | T |
| 8 | DDB0231686 | GO:0009035 | T |
| 9 | DDB0185081 | GO:0005515 | T |
| 9 | DDB0185081 | GO:0003779 | F |
| 10 | DDB0220033 | GO:0016740 | T |
| 11 | DDB0216397 | GO:0003700 | T |
| 11 | DDB0216397 | GO:0003677 | T |
| 12 | DDB0191160 | - | - |
| 13 | DDB0232364 | GO:0004520 | T |
| 13 | DDB0232364 | GO:0000014 | T |
| 14 | DDB0232361 | GO:0004003 | T |
| 14 | DDB0232361 | GO:0016887 | T |
| 14 | DDB0232361 | GO:0008094 | T |
| 14 | DDB0232361 | GO:0005524 | T |
| 14 | DDB0232361 | GO:0008026 | T |
| 14 | DDB0232361 | GO:0003677 | T |
| 14 | DDB0232361 | GO:0003678 | T |
| 14 | DDB0232361 | GO:0003824 | T |
| 14 | DDB0232361 | GO:0003676 | T |
| 14 | DDB0232361 | GO:0004386 | T |
| 15 | DDB0232366 | GO:0005488 | T |
| 16 | DDB0231026 | GO:0004347 | T |
| 16 | DDB0231026 | GO:0016853 | T |
| 17 | DDB0191459 | GO:0003677 | T |
| 17 | DDB0191459 | GO:0003700 | T |
| 18 | DDB0216302 | GO:0003677 | T |
| 18 | DDB0216302 | GO:0003682 | T |
| 19 | DDB0231424 | - | - |
| 20 | DDB0216536 | GO:0003824 | T |
| 20 | DDB0216536 | GO:0016301 | T |
| 20 | DDB0216536 | GO:0004672 | T |
| 20 | DDB0216536 | GO:0016740 | T |
| 20 | DDB0216536 | GO:0005025 | T |
| 20 | DDB0216536 | GO:0004674 | T |
| 20 | DDB0216536 | GO:0004675 | T |
| 20 | DDB0216536 | GO:0005024 | T |
| 20 | DDB0216536 | GO:0004698 | T |
| 20 | DDB0216536 | GO:0004701 | T |
| 20 | DDB0216536 | GO:0004687 | T |
| 20 | DDB0216536 | GO:0016909 | T |
| 20 | DDB0216536 | GO:0005026 | T |
| 20 | DDB0216536 | GO:0004700 | T |
| 20 | DDB0216536 | GO:0004702 | T |
| 20 | DDB0216536 | GO:0004709 | T |
| 20 | DDB0216536 | GO:0004714 | T |
| 20 | DDB0216536 | GO:0004716 | T |
| 20 | DDB0216536 | GO:0005007 | T |
| 20 | DDB0216536 | GO:0005009 | F |
| 21 | DDB0217079 | GO:0005262 | T |
| 22 | DDB0217187 | GO:0003677 | T |
| 22 | DDB0217187 | GO:0003700 | T |
| 23 | DDB0215140 | GO:0003677 | T |
| 23 | DDB0215140 | GO:0003683 | T |
| 23 | DDB0215140 | GO:0003682 | T |
| 24 | DDB0232973 | GO:0016740 | T |
| 24 | DDB0232973 | GO:0003908 | T |
| 24 | DDB0232973 | GO:0008168 | T |
| 25 | DDB0229897 | GO:0003677 | T |
| 25 | DDB0229897 | GO:0003684 | T |
| 25 | DDB0229897 | GO:0005524 | T |
| 26 | DDB0233028 | GO:0003824 | T |
| 27 | DDB0231779 | - | - |
| 28 | DDB0190048 | GO:0003824 | T |
| 28 | DDB0190048 | GO:0004114 | T |
| 28 | DDB0190048 | GO:0016787 | T |
| 28 | DDB0190048 | GO:0004115 | T |
| 28 | DDB0190048 | GO:0004116 | T |
| 28 | DDB0190048 | GO:0004117 | T |
| 28 | DDB0190048 | GO:0004118 | T |
| 28 | DDB0190048 | GO:0004119 | T |
| 29 | DDB0185057 | GO:0003824 | T |
| 29 | DDB0185057 | GO:0016787 | T |
| 29 | DDB0185057 | GO:0004721 | T |
| 29 | DDB0185057 | GO:0016791 | T |
| 29 | DDB0185057 | GO:0008138 | T |
| 29 | DDB0185057 | GO:0004722 | T |
| 29 | DDB0185057 | GO:0017018 | T |
| 30 | DDB0220676 | GO:0003824 | T |
| 31 | DDB0216197 | GO:0003824 | T |
| 31 | DDB0216197 | GO:0016787 | T |
| 31 | DDB0216197 | GO:0004114 | T |
| 31 | DDB0216197 | GO:0004115 | T |
| 31 | DDB0216197 | GO:0004117 | T |
| 31 | DDB0216197 | GO:0004116 | F |
| 32 | DDB0219977 | GO:0003735 | T |
| 33 | DDB0232374 | GO:0005525 | T |
| 33 | DDB0232374 | GO:0019001 | T |
| 33 | DDB0232374 | GO:0003926 | T |
| 34 | DDB0216434 | GO:0003677 | T |
| 35 | DDB0229932 | - | - |
| 36 | DDB0231000 | - | - |
| 37 | DDB0231094 | - | - |
| 38 | DDB0230092 | GO:0003824 | T |
| 38 | DDB0230092 | GO:0016740 | T |
| 38 | DDB0230092 | GO:0004069 | T |
| 38 | DDB0230092 | GO:0008483 | T |
| 39 | DDB0191132 | - | - |
| 40 | DDB0191222 | GO:0005524 | T |
| 40 | DDB0191222 | GO:0015406 | T |
| 40 | DDB0191222 | GO:0005215 | T |
| 40 | DDB0191222 | GO:0000166 | T |
| 40 | DDB0191222 | GO:0015596 | F |
| 40 | DDB0191222 | GO:0015598 | F |
| 40 | DDB0191222 | GO:0015613 | T |
| 40 | DDB0191222 | GO:0015421 | F |
| 40 | DDB0191222 | GO:0015424 | F |
| 40 | DDB0191222 | GO:0015412 | T |
| 40 | DDB0191222 | GO:0015611 | T |
| 40 | DDB0191222 | GO:0015415 | T |
| 40 | DDB0191222 | GO:0015417 | F |
| 40 | DDB0191222 | GO:0015419 | F |
| 40 | DDB0191222 | GO:0015420 | F |
| 40 | DDB0191222 | GO:0008559 | F |
| 40 | DDB0191222 | GO:0015430 | F |
| 40 | DDB0191222 | GO:0005395 | F |
| 40 | DDB0191222 | GO:0015423 | T |
| 40 | DDB0191222 | GO:0004009 | T |
| 41 | DDB0191133 | GO:0005488 | T |
| 41 | DDB0191133 | GO:0005509 | T |
| 41 | DDB0191133 | GO:0005200 | T |
| 42 | DDB0231684 | GO:0003824 | T |
| 42 | DDB0231684 | GO:0016874 | T |
| 42 | DDB0231684 | GO:0016877 | T |
| 42 | DDB0231684 | GO:0003987 | T |
| 42 | DDB0231684 | GO:0016878 | T |
| 42 | DDB0231684 | GO:0004467 | T |
| 42 | DDB0231684 | GO:0016881 | T |
| 42 | DDB0231684 | GO:0016405 | T |
| 43 | DDB0220444 | GO:0005200 | T |
| 43 | DDB0220444 | GO:0005198 | T |
| 44 | DDB0230097 | GO:0004017 | T |
| 44 | DDB0230097 | GO:0016740 | T |
| 45 | DDB0220136 | - | - |
| 46 | DDB0231341 | - | - |
| 47 | DDB0201588 | - | - |
| 48 | DDB0214894 | GO:0004813 | T |
| 48 | DDB0214894 | GO:0016874 | T |
| 48 | DDB0214894 | GO:0004812 | T |
| 49 | DDB0231453 | - | - |
| 50 | DDB0215363 | GO:0016491 | T |
| 50 | DDB0215363 | GO:0004032 | T |
| 50 | DDB0215363 | GO:0004033 | T |
| 50 | DDB0215363 | GO:0008106 | T |
| 51 | DDB0231275 | - | - |
| 52 | DDB0191089 | GO:0016787 | T |
| 52 | DDB0191089 | GO:0003876 | T |
| 52 | DDB0191089 | GO:0016814 | T |
| 52 | DDB0191089 | GO:0019239 | T |
| 53 | DDB0201558 | GO:0005471 | T |
| 53 | DDB0201558 | GO:0005215 | T |
| 53 | DDB0201558 | GO:0005515 | T |
| 53 | DDB0201558 | GO:0005386 | T |
| 53 | DDB0201558 | GO:0005488 | T |
| 53 | DDB0201558 | GO:0005509 | T |
| 54 | DDB0214928 | - | - |
| 55 | DDB0231277 | - | - |
| 56 | DDB0191101 | GO:0005525 | T |
| 56 | DDB0191101 | GO:0003925 | T |
| 56 | DDB0191101 | GO:0003924 | T |
| 56 | DDB0191101 | GO:0019001 | T |
| 56 | DDB0191101 | GO:0003928 | T |
| 56 | DDB0191101 | GO:0003926 | T |
| 56 | DDB0191101 | GO:0003824 | T |
| 56 | DDB0191101 | GO:0003927 | T |
| 56 | DDB0191101 | GO:0003930 | T |
| 56 | DDB0191101 | GO:0003931 | T |
| 57 | DDB0229363 | GO:0016301 | T |
| 57 | DDB0229363 | GO:0004672 | T |
| 57 | DDB0229363 | GO:0016740 | T |
| 57 | DDB0229363 | GO:0004714 | T |
| 57 | DDB0229363 | GO:0004681 | F |
| 57 | DDB0229363 | GO:0005024 | F |
| 57 | DDB0229363 | GO:0004698 | F |
| 57 | DDB0229363 | GO:0004701 | F |
| 57 | DDB0229363 | GO:0005025 | F |
| 57 | DDB0229363 | GO:0004702 | F |
| 57 | DDB0229363 | GO:0004705 | T |
| 57 | DDB0229363 | GO:0004675 | F |
| 57 | DDB0229363 | GO:0016909 | F |
| 57 | DDB0229363 | GO:0017002 | T |
| 57 | DDB0229363 | GO:0005026 | F |
| 57 | DDB0229363 | GO:0004691 | T |
| 57 | DDB0229363 | GO:0004692 | T |
| 57 | DDB0229363 | GO:0004709 | T |
| 57 | DDB0229363 | GO:0004716 | T |
| 57 | DDB0229363 | GO:0005003 | T |
| 58 | DDB0229368 | GO:0003924 | T |
| 58 | DDB0229368 | GO:0005525 | T |
| 58 | DDB0229368 | GO:0003925 | T |
| 58 | DDB0229368 | GO:0019001 | T |
| 58 | DDB0229368 | GO:0003928 | T |
| 58 | DDB0229368 | GO:0003926 | T |
| 58 | DDB0229368 | GO:0003931 | T |
| 59 | DDB0191175 | - | - |
| 60 | DDB0214955 | GO:0005509 | T |
| 60 | DDB0214955 | GO:0005488 | T |
| 61 | DDB0185021 | GO:0016787 | T |
| 61 | DDB0185021 | GO:0004721 | T |
| 61 | DDB0185021 | GO:0016791 | T |
| 61 | DDB0185021 | GO:0004722 | T |
| 61 | DDB0185021 | GO:0000158 | T |
| 61 | DDB0185021 | GO:0004723 | T |
| 61 | DDB0185021 | GO:0017018 | T |
| 61 | DDB0185021 | GO:0004724 | T |
| 61 | DDB0185021 | GO:0030357 | T |
| 61 | DDB0185021 | GO:0000163 | F |
| 61 | DDB0185021 | GO:0015071 | T |
| 61 | DDB0185021 | GO:0008420 | T |
| 62 | DDB0220110 | GO:0003824 | T |
| 62 | DDB0220110 | GO:0016787 | T |
| 62 | DDB0220110 | GO:0016788 | T |
| 62 | DDB0220110 | GO:0016298 | T |
| 62 | DDB0220110 | GO:0016789 | T |
| 62 | DDB0220110 | GO:0004806 | T |
| 63 | DDB0215368 | - | - |
| 64 | DDB0215007 | GO:0004872 | T |
| 64 | DDB0215007 | GO:0004930 | T |
| 64 | DDB0215007 | GO:0004967 | T |
| 64 | DDB0215007 | GO:0004888 | T |
| 64 | DDB0215007 | GO:0008528 | T |
| 65 | DDB0230136 | - | - |
| 66 | DDB0191384 | GO:0005509 | T |
| 66 | DDB0191384 | GO:0005514 | T |
| 67 | DDB0191103 | GO:0005515 | T |
| 67 | DDB0191103 | GO:0003779 | T |
| 68 | DDB0185191 | - | - |
| 69 | DDB0191386 | GO:0003824 | T |
| 69 | DDB0191386 | GO:0016740 | T |
| 69 | DDB0191386 | GO:0008415 | T |
| 70 | DDB0191314 | - | - |
| 71 | DDB0191318 | GO:0016829 | T |
| 71 | DDB0191318 | GO:0004121 | T |
| 71 | DDB0191318 | GO:0004123 | T |
| 72 | DDB0191434 | - | - |
| 73 | DDB0191159 | - | - |
| 74 | DDB0232045 | - | - |
| 75 | DDB0233144 | - | - |
| 76 | DDB0231400 | GO:0003824 | T |
| 76 | DDB0231400 | GO:0016829 | T |
| 76 | DDB0231400 | GO:0004333 | T |
| 76 | DDB0231400 | GO:0004056 | T |
| 76 | DDB0231400 | GO:0008797 | T |
| 77 | DDB0219943 | GO:0016740 | T |
| 77 | DDB0219943 | GO:0008417 | T |
| 77 | DDB0219943 | GO:0016757 | T |
| 77 | DDB0219943 | GO:0017083 | T |
| 77 | DDB0219943 | GO:0017060 | T |
| 77 | DDB0219943 | GO:0018392 | T |
| 78 | DDB0231448 | GO:0016740 | T |
| 78 | DDB0231448 | GO:0003824 | T |
| 78 | DDB0231448 | GO:0008483 | T |
| 78 | DDB0231448 | GO:0016769 | T |
| 78 | DDB0231448 | GO:0003867 | T |
| 78 | DDB0231448 | GO:0003992 | T |
| 78 | DDB0231448 | GO:0008453 | T |
| 78 | DDB0231448 | GO:0004015 | T |
| 79 | DDB0231446 | GO:0016829 | T |
| 79 | DDB0231446 | GO:0004351 | T |
| 79 | DDB0231446 | GO:0016831 | T |
| 80 | DDB0231575 | GO:0003824 | T |
| 80 | DDB0231575 | GO:0016853 | T |
| 80 | DDB0231575 | GO:0003978 | T |
| 80 | DDB0231575 | GO:0016857 | T |
| 81 | DDB0231439 | GO:0016829 | T |
| 81 | DDB0231439 | GO:0004351 | T |
| 81 | DDB0231439 | GO:0016831 | T |
| 82 | DDB0231667 | GO:0004335 | T |
| 82 | DDB0231667 | GO:0016740 | T |
| 83 | DDB0231403 | GO:0016874 | T |
| 83 | DDB0231403 | GO:0004357 | T |
| 84 | DDB0231130 | GO:0004375 | T |
| 84 | DDB0231130 | GO:0016491 | T |
| 85 | DDB0231218 | GO:0003824 | T |
| 85 | DDB0231218 | GO:0016740 | T |
| 86 | DDB0231642 | GO:0016787 | T |
| 86 | DDB0231642 | GO:0004553 | T |
| 86 | DDB0231642 | GO:0016798 | T |
| 86 | DDB0231642 | GO:0008810 | T |
| 87 | DDB0231714 | GO:0016491 | T |
| 88 | DDB0231727 | GO:0016829 | T |
| 89 | DDB0231738 | - | - |
| 90 | DDB0230211 | GO:0016787 | T |
| 90 | DDB0230211 | GO:0008892 | T |
| 91 | DDB0201569 | GO:0016787 | T |
| 91 | DDB0201569 | GO:0016798 | T |
| 91 | DDB0201569 | GO:0004559 | T |
| 92 | DDB0230181 | GO:0003824 | T |
| 92 | DDB0230181 | GO:0016829 | T |
| 92 | DDB0230181 | GO:0004474 | T |
| 93 | DDB0232403 | GO:0005488 | T |
| 93 | DDB0232403 | GO:0016787 | T |
| 94 | DDB0231478 | GO:0003824 | T |
| 94 | DDB0231478 | GO:0016740 | T |
| 94 | DDB0231478 | GO:0008483 | T |
| 94 | DDB0231478 | GO:0016769 | T |
| 94 | DDB0231478 | GO:0003992 | T |
| 94 | DDB0231478 | GO:0004587 | T |
| 94 | DDB0231478 | GO:0008453 | T |
| 94 | DDB0231478 | GO:0003867 | T |
| 94 | DDB0231478 | GO:0004015 | T |
| 94 | DDB0231478 | GO:0009016 | T |
| 95 | DDB0231870 | GO:0016758 | T |
| 95 | DDB0231870 | GO:0003824 | T |
| 95 | DDB0231870 | GO:0016757 | T |
| 95 | DDB0231870 | GO:0016740 | T |
| 96 | DDB0185225 | - | - |
| 97 | DDB0232978 | - | - |
| 98 | DDB0231224 | GO:0004730 | T |
| 98 | DDB0231224 | GO:0016829 | T |
| 99 | DDB0214958 | GO:0003824 | T |
| 99 | DDB0214958 | GO:0004590 | T |
| 99 | DDB0214958 | GO:0004588 | T |
| 99 | DDB0214958 | GO:0016740 | T |
| 99 | DDB0214958 | GO:0016829 | T |
| 99 | DDB0214958 | GO:0016757 | T |
| 100 | DDB0191479 | GO:0000156 | T |
| 100 | DDB0191479 | GO:0004114 | T |
